# Bronchoscopy versus an endotracheal tube mounted camera for the peri-interventional visualization of percutaneous dilatational tracheostomy - a prospective, randomized trial (VivaPDT)

**DOI:** 10.1186/s13054-017-1901-0

**Published:** 2017-12-29

**Authors:** Jörn Grensemann, Lars Eichler, Sophie Kähler, Dominik Jarczak, Marcel Simon, Hans O. Pinnschmidt, Stefan Kluge

**Affiliations:** Department of Intensive Care Medicine, University Medical Center Hamburg-Eppendorf, Martinistraße 52, 20246 Hamburg, Germany

**Keywords:** Tracheostomy, Diagnostic equipment, Diagnostic techniques, Respiratory system, Bronchoscopy, Hypercapnia, Respiration, Artificial, Ventilator weaning, Intubation, Intratracheal, Airway management, Critical care

## Abstract

**Background:**

Percutaneous dilatational tracheostomy (PDT) in critically ill patients often involves bronchoscopic optical guidance. However, this procedure is not without disadvantages. Therefore, we aimed to study a recently introduced endotracheal tube-mounted camera (VivaSight^TM^-SL tube [VST]; ETView, Misgav, Israel) for guiding PDT.

**Methods:**

This was a randomized controlled trial involving 46 critically ill patients who received PDT using optical guidance with a VST or with bronchoscopy. The primary outcome measure was visualization of the tracheal structures (i.e., identification and monitoring of the thyroid, cricoid, and tracheal cartilage and the posterior wall) rated on 4-point Likert scales. Secondary measures were the quality of ventilation (before puncture and during the tracheostomy procedure rated on 4-point Likert scales) and blood gases sampled at standardized time points.

**Results:**

The mean ratings for visualization (lower values better; values given for per-protocol analysis) were 5.4 (95% CI 4.5–6.3) for the VST group and 4.0 (95% CI 4.0–4.0) for the bronchoscopy group (*p* < 0.001). Mean ventilation ratings were 2.5 (95% CI 2.1–2.9) for VST and 5.0 (95% CI 4.4–5.7) for bronchoscopy (*p* < 0.001). Arterial carbon dioxide increased to 5.9 (95% CI 5.4–6.5) kPa in the VST group vs. 8.3 (95% CI 7.2–9.5) kPa in the bronchoscopy group (*p* < 0.001), and pH decreased to 7.40 (95% CI 7.36–7.43) in the VST group vs. 7.26 (95% CI 7.22–7.30) in the bronchoscopy group (*p* < 0.001), at the end of the intervention.

**Conclusions:**

Visualization of PDT with the VST is not noninferior to guidance by bronchoscopy. Ventilation is superior with less hypercarbia with the VST. Because visualization is not a prerequisite for PDT, patients requiring stable ventilation with normocarbia may benefit from PDT with the VST.

**Trial registration:**

ClinicalTrials.gov, NCT02861001. Registered on 13 June 2016.

**Electronic supplementary material:**

The online version of this article (doi:10.1186/s13054-017-1901-0) contains supplementary material, which is available to authorized users.

## Background

Critically ill patients requiring long-term ventilation often receive a tracheostomy to facilitate weaning from the ventilator, reduce dead space, and avoid laryngeal injury [1–3]. Percutaneous dilatational tracheostomy (PDT) has gained widespread acceptance and has replaced surgical tracheostomy as the technique of choice in many intensive care units for its convenience and speed [4]. Most percutaneous methods use a modification of the Seldinger technique that involves the puncture of the trachea, the introduction of a guidewire, and the dilation of the tracheostomy tract. Bronchoscopic guidance of the PDT is frequently used as a safety measure facilitating the choice of the correct tracheostomy site, verification of the intratracheal guidewire and dilator placements, and positioning of the tracheal cannula [5]. Bronchoscopic guidance may also minimize the risk of complications, especially posterior tracheal wall injury [6, 7]. However, the role of bronchoscopy is controversial because it can lead to carbon dioxide retention and hypoxia, as well as because it increases procedural time, cost, and the complexity of PDT [8]. In recent years, the role of bronchoscopy in PDT has been questioned, and the use of neck ultrasound has been suggested [9]. It has been shown that ultrasound is not inferior to bronchoscopy in terms of major complications and procedure duration, but this technique does not provide a view of the posterior wall of the trachea [10].

An endotracheal tube with an integrated camera at its tip is now available that permits continuous visualization of the larynx and trachea on a monitor connected to the camera (VivaSight^TM^-SL [VST]; ETView Ltd., Misgav, Israel) [11]. The camera is laminated into the anterior wall of the tube and equipped with a rinsing channel. As we have shown before, PDT with this tube is a feasible alternative to bronchoscopic guidance and might improve patients’ gas exchange, airway pressures, and ventilation compared with PDT with bronchoscopic guidance [12]. Because one of the main goals of visual guidance is to identify the correct point of puncture and the posterior wall of the trachea, we assessed visualization during PDT via the VST as well as changes in patients’ gas exchange and pH values against bronchoscopy in a prospective, randomized, noninferiority study.

## Methods

### Study design

The VivaPDT trial was a prospective, randomized study conducted in the Department of Intensive Care Medicine at the University Medical Center Hamburg-Eppendorf, Germany. Patients were eligible if they were at least 18 years old and had received PDT for long-term ventilation and if written informed consent was obtained from a legal guardian. We sought to enroll 46 patients who were randomized (using sealed, opaque envelopes) in a 1:1 ratio to PDT with optical guidance by VST or by bronchoscopy. An a priori power analysis for noninferiority testing indicated that a sample size of 46 would be sufficient to detect a difference of 20% in the visualization score (noninferiority margin 0.8) with error probabilities of α = 0.05 and 1 − β = 0.80 (Power Analysis and Sample Size [PASS] version 08.0.6 software; NCSS, Kaysville, UT, USA). A difference of 20% was deemed clinically acceptable and chosen arbitrarily owing to a lack of data in the literature. The institutional research ethics board approved the protocol. The study is registered with ClinicalTrials.gov (NCT02861001).

### Tracheostomy

All patients underwent PDT with the Ciaglia single-step dilator technique (Ciaglia Blue Rhino^®^ G2; Cook Medical, Bloomington, IN, USA) [13]. Before the intervention, patients were mechanically ventilated in a pressure-controlled mode (bilevel positive airway pressure, Evita V500, Drägerwerk, Lübeck, Germany) via an orally placed endotracheal tube. Patients were anesthetized with propofol and/or midazolam and sufentanil. Rocuronium was used for muscle relaxation. A brief description of our tracheostomy protocol has been published before [13].

Direct laryngoscopy was performed, and the view of the larynx was assessed, according to the method of Cormack and Lehane [14]. To reduce the risk of airway complications during and following tracheostomy, patients with a Cormack and Lehane score ≥ 3 were excluded and received surgical tracheostomy. This exclusion was enforced according to the hospital protocol for patients receiving PDT and ensured that in cases of an accidental extubation during tracheostomy, the airway could easily be reestablished under direct laryngoscopy. For this purpose, a laryngoscope was always readily available throughout the procedure. The trachea was cannulated between the second and third tracheal cartilage after an optional blunt dissection of the subcutaneous tissue. Tracheostomies were performed by senior physicians with a specialization in intensive care medicine and an experience of at least 30 PDTs.

### Endotracheal tube-mounted camera procedure

In patients randomized to the VST group, the endotracheal tube was exchanged for a VST with an inner diameter of 7.5 mm for female patients or 8.0 mm for male patients under direct laryngoscopy, after thorough aspiration of all secretions and after preoxygenation with 100% oxygen. A swivel connector was added to the airway tubing for conventional suctioning of secretions from the trachea if necessary. The tube-mounted camera was connected to a VivaSight™-Max monitor (ETView Ltd.), which was attached to the bed rails. The VST was retracted until the cricoid cartilage was visible, manipulated for an optimal view of the procedure, and held in this position by an assistant. For patients randomized to the VST group, a bronchoscope was available as a backup safety measure and could be used at the treating physician’s discretion.

### Bronchoscopic procedure

In patients randomized to the bronchoscopy group, the endotracheal tube was retracted until the cricoid cartilage was visible under optical guidance using a bronchoscope (Olympus BF-P60; Olympus Medical Systems Corp., Tokyo, Japan) connected to a monitor (Olympus Medical Systems Corp.).

### Outcome parameters

During PDT, visualization of the trachea and quality of ventilation were rated according to a score previously used by our study group (*see* Table 1) [12]. The score was based on a modification of the scoring of Linstedt et al. [15]. Each item was rated on a 4-point Likert scale as follows: 1 = very good; 2 = good; 3 = difficult; or 4 = impossible [16]. The quality of ventilation (Table 1, line E) was rated twice: The first rating was obtained before puncture of the trachea (E1), and the second rating reflected the worst ventilation during the PDT (E2). To reduce a potential bias introduced during scoring, all ratings were obtained by an independent physician who observed the PDT but did not participate in the intervention.

**Table 1:** Rating scale for the visualization of tracheal structures and ventilation during percutaneous dilatational tracheostomy

	Rating	1	2	3	4
A	Identification of thyroid cartilage, cricoid cartilage, first to third tracheal cartilage	Reliable identification	Only cricoid cartilage and tracheal cartilages	Only tracheal cartilage	No vision of tracheal structures
B	Visualization of tracheal circumference	Complete circumference	One-third to two-thirds of circumference	Only small parts of trachea	No vision of tracheal structures
C	Monitoring puncture midline + level below first or second tracheal cartilage	Reliable identification	Midline can be displayed, level uncertain, but below the first tracheal cartilage	Level of puncture uncertain	No vision of tracheal structures
D	Monitoring dilatation anterior wall and pars membranacea (p.m.) visible	Reliable identification	p.m. only	Only small parts of trachea visible, no control of p.m.	No vision of tracheal structures
E	Quality of ventilation before puncture and worst ventilation during PDT, respectively	Minute ventilation (MV) as before starting PDT	MV < 2 L/minute or SpO_2_ 80–90% (>2 minutes)	MV < 0.5 L/minute or SpO_2_ 70–79% (> 2 minutes)	MV = 0 or SpO_2_ < 70% (> 2 minutes)

*Abbreviations: MV* Minute ventilation; *PDT* Percutaneous dilatational tracheostomy; *p.m.* Pars membranacea of the trachea, *SpO*_*2*_ Oxygen saturation as measured by pulse oximetry

From Grensemann et al. [12] (modified from Linstedt et al. [15])

Rating system: 1 = very good; 2 = good; 3 = difficult; 4 = impossible. The quality of ventilation (line E) was rated twice (i.e., before puncture [E1] and to reflect the worst ventilation during tracheostomy [E2])

To assess partial pressure of arterial oxygen (PaO_2_), partial pressure of arterial carbon dioxide (PaCO_2_), and pH values, arterial blood gas (ABG) values were obtained before skin incision (time point 2) and immediately after insertion of the tracheal cannula (time point 3). ABG values prior to the start of the intervention (time point 1) were obtained from the patients’ electronic medical records (Integrated Care Manager ICM version 8.12; Drägerwerk).

Minute ventilation (MV) during tracheostomy, blood pressure, oxygen saturation by pulse oximetry, and capnography (Infinity Delta vital signs monitor; Drägerwerk) were recorded in addition to patients’ demographic parameters and the duration of the intervention. The Acute Physiology and Chronic Health Evaluation II score [17] and the Sequential Organ Failure Assessment score [18] were recorded on the day of examination as measures of disease severity.

The primary endpoint was the quality of visualization as measured by items A through D on the score. Secondary endpoints were the quality of ventilation (scoring items E1 and E2); changes in PaCO_2_, pH, end-tidal carbon dioxide, and PaO_2_; duration of intervention; and adverse events related to PDT within 1 week of the intervention.

### Statistics

Microsoft Excel 2016 software (Microsoft Corp., Redmond, WA, USA) was used for data management, and the IBM SPSS Statistics software package (version 23; IBM, Armonk, NY, USA) was used for statistical analysis. We used Welch tests for comparisons of scores. Visualization and ventilation scores were tested for noninferiority of VivaSight compared with bronchoscopy. Noninferiority was considered established if the upper limit of the 95% CI of the difference between the scores of the VivaSight and bronchoscopy group of the respective outcome variable did not surpass the mean of the score of the bronchoscopy group by 20% or more (lower scores indicate better performance). The 95% CIs of the mean of the scores were calculated as mean plus and minus the respective value of the *t*-distribution multiplied by the SEM calculated as the SD divided by the square root of the sample size. We used linear mixed models with post hoc pairwise comparisons of estimated marginal means for hemodynamic and respiratory variables. In the mixed model analyses, fixed effects of the treatment groups, time points and group × time point, and random intercepts for patients were assumed, employing a variance component covariance matrix. We performed both intention-to-treat (ITT) and per-protocol (PP) analyses. Two-tailed *p* values < 0.05 were regarded as statistically significant.

## Results

**Fig. 1:**
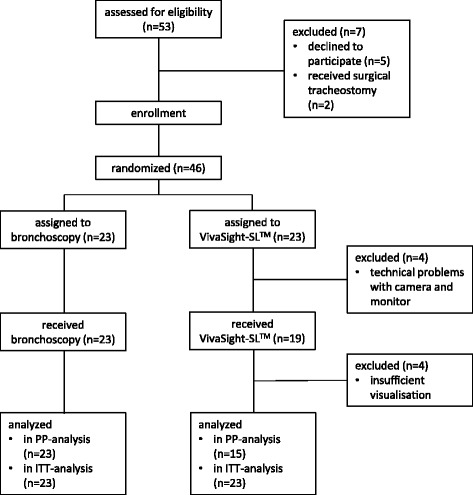
Consolidated Standards of Reporting Trials (CONSORT) diagram. *PP* Per protocol, *ITT* Intention to treat

From June 2016 to January 2017, a total of 46 patients receiving PDT for prolonged mechanical ventilation were randomized to either VST or bronchoscopy in a 1:1 ratio (*see* Fig. 1). Patients’ baseline characteristics are shown in Table 2.

**Table 2:** Patient characteristics

	VivaSight (*n* = 23)	Bronchoscopy (*n* = 23)
Age, years	62 ± 14	63 ± 15
Sex	Male: 14 (61%)	Male: 13 (57%)
	Female: 9 (39%)	Female: 10 (43%)
SOFA score	11 (8–12)	10 (8–11)
APACHE II score	23 (18–30)	25 (22–33)

*Abbreviations: SOFA* Sequential Organ Failure Assessment, *APACHE II* Acute Physiology and Chronic Health Evaluation II

Data are shown as the mean ± SD or the median and IQR in parentheses as appropriate

All patients had a Cormack and Lehane score of 1 or 2. The mean procedure duration from skin incision to insertion of the tracheal cannula did not differ significantly (n.s.) between the groups, being 14.7 ± 11.0 minutes in the VST group vs. 10.6 ± 8.2 minutes in the bronchoscopy group in the ITT analysis, and 10.0 ± 4.8 minutes vs. 10.6 ± 8.2 minutes (n.s.) in the PP analysis, respectively.

**Fig. 2:**
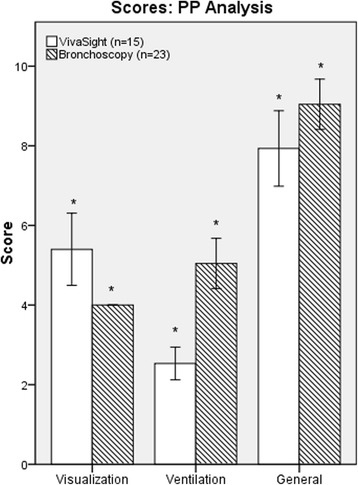
Rating of visualization and ventilation according to score (per-protocol analysis). Lower scores indicate better performance. *PP* Per protocol

Noninferiority for visualization (the primary endpoint) could not be demonstrated in the VST group. Mean visualization scores were 5.9 (95% CIs for the mean 4.7–7.1) for the VST group vs. 4.0 (4.0–4.0) for the bronchoscopy group with a mean difference of 1.9 (0.7–3.1) in the ITT analysis and 5.4 (4.5–6.3) vs. 4.0 (4.0–4.0), mean difference 1.4 (0.5–2.3), in the PP analysis, respectively (*see* Fig. 2; lower values indicate better visualization). Ventilation was rated 2.8 (2.3–3.3) for the VST group vs. 5.0 (4.4–5.7) for the bronchoscopy group, mean difference −2.3 (−3.0 to −1.5) in the ITT analysis and 2.5 (2.1–2.9) vs. 5.0 (4.4–5.7), mean difference −2.5 (−3.2 to −1.8) in the PP analysis (*see* Fig. 2). An additional figure showing the ITT analysis is presented in Additional file 1. For score item A (identification of thyroid cartilage, cricoid cartilage, first to third tracheal cartilage) and item C (monitoring puncture: midline + level below first or second tracheal cartilage), we found no significant difference between the groups. For item B (visualization of tracheal circumference), the VST group was inferior to the bronchoscopy group and for item D (monitoring dilatation: anterior wall and pars membranacea visible), no noninferiority could be established for the VST group. For the items evaluating ventilation (items E1 and E2), the VST group was superior to the bronchoscopy group. An overview on the score analyses is presented in Additional file 2.

**Fig. 3:**
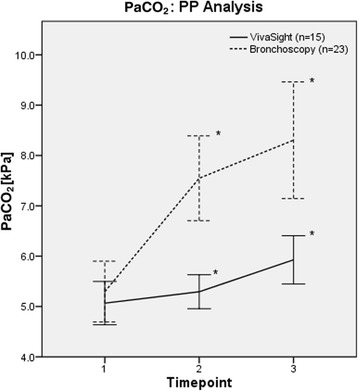
Changes in partial pressure of arterial carbon dioxide (PaCO_2_) (per-protocol [PP] analysis). Time point 1 = before start of intervention; time point 2 = before tracheal cannulation; time point 3 = after insertion of tracheal cannula. * *p* < 0.05 for difference between groups at respective time points (*t* test).

**Fig. 4:**
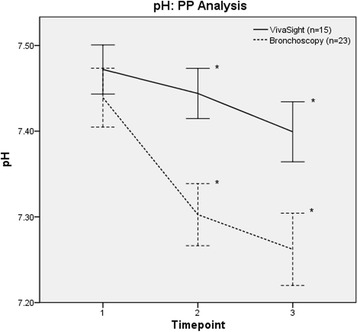
Changes in pH (per-protocol [PP] analysis). Time point 1 = before start of intervention; time point 2 = before tracheal cannulation; time point 3 = after insertion of tracheal cannula. * *p* < 0.05 for difference between groups at respective time points (*t* test).

In the VST group, mean PaCO_2_ was lower than in the bronchoscopy group before (5.3 [5.0–5.7] vs. 7.6 [6.7–8.4] kPa, *p* < 0.001, PP analysis) and after insertion of the tracheal cannula (5.9 [5.4–6.5] vs. 8.3 [7.2–9.5] kPa, *p* < 0.001) (*see* Fig. 3). Conversely, pH values were higher in the VST group before puncture (7.44 [7.41–7.47] vs. 7.30 [7.27–7.34], *p* < 0.001) (*see* Fig. 4) and after insertion of the tracheal cannula (7.40 [7.36–7.43] vs. 7.26 [7.22–7.30], *p* < 0.001). MV was higher in the VST group (6.7 [5.3–8.2] vs. 3.5 [2.5–4.8] L/minute, *p* = 0.002) before puncture. An overview of arterial blood gas analyses and respiratory parameters is given in Table 3 (PP analysis). Values for the ITT analysis are available Additional files 1, 3, 4, and 5.

**Table 3:** Arterial blood gas analyses and respiratory values (per-protocol analysis)

	Time point 1		Time point 2			Time point 3		
	VivaSight™	Bronchoscopy	VivaSight™	Bronchoscopy	*p* Value	VivaSight™	Bronchoscopy	*p* Value
pH	7.47 (7.44–7.50)	7.44 (7.40–7.47)	7.44 (7.41–7.47)	7.30 (7.27–7.34)^a^	< 0.001	7.40 (7.36–7.43)^a,b^	7.26 (7.22–7.30)^a,b^	< 0.001
PaO_2_, kPa	10.7 (10.1–11.3)	11.0 (10.2–11.9)	54.3 (45.7–62.9)^a^	52.6 (44.7–60.5)^a^	n.s.	50.8 (41.2–60.3)^a^	51.3 (43.5–59.1)^a^	n.s.
PaCO_2_, kPa	5.1 (4.6–5.6)	5.3 (4.7–5.9)	5.3 (5.0–5.7)	7.6 (6.7–8.4)^a^	< 0.001	5.9 (5.4–6.5)	8.3 (7.2–9.5)^a,b^	< 0.001
Lactate, mmol/L	0.9 (0.6–1.1)	1.0 (0.7–1.3)	0.8 (0.6–1.0)	0.9 (0.6–1.2)	n.s.	0.8 (0.6–1.0)^a^	0.8 (0.6–1.1)	n.s.
Hb, g/dl	8.8 (8.1–9.4)	9.0 (8.4–9.5)	8.7 (7.9–9.4)	8.9 (8.3–9.6)	n.s.	8.6 (8.0–9.3)	9.0 (8.3–9.6)	n.s.
SaO_2_, %	97 (96–98)	96 (96–97)	100 (100–100)^a^	100 (100–100)	n.s.	100 (100–100)^a^	100 (100–100)	n.s.
Paw, hPa	21 (18–24)	21 (19–23)	22 (19–24)	23 (21–26)	n.s.	21 (18–25)	23 (21–25)	n.s.
PEEP, hPa	7 (6–8)	7 (6–8)	7 (5–8)	7 (6–7)	n.s.	7 (6–8)	7 (6–7)	n.s.
etCO_2_, kPa	4.9 (4.4–5.4)	4.6 (4.1–5.1)	4.7 (4.2–5.2)	4.5 (3.6–5.4)	n.s.	5.3 (4.8–5.8)	6.4 (5.8–7.1)^a,b^	0.01
V_t_, ml	468 (388–549)	446 (386–512)	522 (295–749)	250 (148–371)^a^	0.001	390 (301 –478)	380 (257–465)	n.s.
MV, L/minute	8.0 (7.2–8.9)	9.6 (8.0–11.3)	6.7 (5.3–8.2)	3.5 (2.5–4.8)^a^	0.002	7.9 (6.0–9.8)	6.3 (4.8–7.8)^a,b^	n.s.
C, ml/hPa	54 (37–71)	57 (41–72)	31 (22–41)^a^	24 (17–34)^a^	n.s.	45 (25–65)	29 (22–38)^a^	n.s.
RR, breaths/minute	18 (16–21)	20 (17–23)	19 (17–21)	20 (18–22)	n.s.	20 (17–22)	21 (18–23)	n.s.
FiO_2_	0.27 (0.23–0.30)	0.32 (0.25–0.39)	0.99 (0.96–1.02)^a^	0.99 (0.97–1.01)^a^	n.s.	0.99 (0.96–1.02)^a^	0.99 (0.97–1.01)^a^	n.s.
SpO_2_, %	98 (97–99)	96 (95–98)	100 (100–100)^a^	100 (99–100)^a^	n.s.	100 (99–100)^a^	99 (99–100)^a^	n.s.
MAP, mmHg	82 (73–92)	83 (76–90)	87 (79–95)	81 (74–88)	n.s.	88 (81–95)	86 (81–93)	n.s.

*Abbreviations: PaO*_*2*_ Partial pressure of arterial oxygen, *PaCO*_*2*_ Partial pressure of arterial carbon dioxide, *SaO*_*2*_ Arterial oxygen saturation, *Paw* Airway pressure, *PEEP* Positive end-expiratory pressure, *etCO*_*2*_ End-tidal carbon dioxide tension, *V*_*t*_ Tidal volume, *MV* Minute ventilation, *C* Compliance, *RR* Respiratory rate, *FiO*_*2*_ Fraction of inspired oxygen, *SpO*_*2*_ Oxygen saturation as measured by pulse oximetry, *MAP* Mean arterial pressure, *Hb* Hemoglobin, n.s. Not statistically significant

Data are shown as the mean and 95% confidence intervals, time point 1: before start of intervention, time point 2: before tracheal cannulation, time point 3: after insertion of tracheal cannula. Statistical analysis was done with linear mixed models. *p* Values in columns indicate differences between VivaSight and bronchoscopy groups at the respective time points

^a^ *p* < 0.05 vs. time point 1

^b^ *p* < 0.05 vs. time point 2

Four patients randomized to the VST group did not receive the allocated intervention owing to various technical problems: camera failure, monitor failure, defective contact between the plug of the camera cable and the monitor cable, and impossibility of clearing the camera of bronchial secretions (each occurring once). In a further four patients in the VST group, the assigned intervention was discontinued owing to incomplete visualization of the tracheal posterior wall and safety concerns of the treating physician. Therefore, PDT was completed under VST optical guidance in only 15 patients (65%) of the VST group. All patients not receiving or completing PDT with VST optical guidance received bronchoscopic guidance. In the bronchoscopy group, all patients received the assigned intervention, and PDT was completed with bronchoscopic guidance. There were two adverse events in the bronchoscopy group (tube dislodgement during intervention, pneumothorax after intervention) and one in the VST group (puncture of the VST tube cuff) (n.s.).

## Discussion

In this noninferiority trial comparing optical guidance by a tube-mounted camera (VST) for PDT with direct bronchoscopy, we found visualization of tracheal structures using VST not to be noninferior to visualization with bronchoscopy according to the scoring system used. However, ventilation was not only noninferior but even superior with less hypercarbia with the VST.

Until now, VST has shown promising results for endotracheal intubation via supraglottic airway devices [11, 19], in manikins [20–23], and in a cadaver study [24]. Furthermore, the feasibility of VST guidance of PDT has been shown recently [12].

Optical guidance in PDT allows for real-time guidance of tracheal cannulation and aids the identification of the correct site of puncture. It is also adopted as a safety measure to prevent damage to the posterior tracheal wall. Although overall visualization with VST was not noninferior to bronchoscopy according to our scoring system, the correct site of cannulation could be identified with VST, which is one of the major goals of optical guidance. When the VST was retracted during the intervention, the camera tended to point at the anterior tracheal wall, allowing the identification of the tracheal cartilages and hence the correct point of cannulation while moving the posterior wall out of the camera’s angle of view. It was sometimes possible to get a view of the posterior wall by manipulating the orientation of the tube, as we have described previously [12]. Visualization of only the anterior wall during cannulation presumably reduces the ability of optical guidance to prevent damage to the posterior tracheal wall, which is the second target of optical guidance. Nevertheless, visualization of the posterior wall is not a prerequisite for PDT [25], which may be performed safely simply by orientation based on anatomical landmarks [26], although some authors recommend the routine use of bronchoscopic guidance [5, 27] to prevent complications. Recently, ultrasound guidance has been evaluated for use during PDT [28]. Using this technique, no image of the posterior wall can be obtained, owing to air in the trachea preventing the ultrasound waves reaching the pars membranacea. Nevertheless, ultrasound-guided PDT was not found to be inferior to bronchoscopy-guided PDT in terms of the occurrence of major complications (i.e., hemodynamic instability, hypoxemia, anatomical injuries, and bleeding) in a randomized controlled noninferiority trial [10]. Researchers in another randomized trial compared the rate of complications between fiberoptic and ultrasound-guided PDT and found the rate of hemorrhage to be lower in the ultrasound-guided group [29], probably owing to the demonstration of blood vessels in the intended puncture path and a subsequent change of path [30]. No damage to the posterior wall occurred in either the ultrasound group or the bronchoscopy group. However, no monitoring for late complications was done in either study. Combining both trials, approximately 200 patients were studied, which may not be sufficient to detect rare complications such as damage to the posterior wall [31].

In our study, in the VST group, we observed ventilation superior to that in the bronchoscopy group. For the bronchoscopy group, PaCO_2_ values increased from the beginning of the intervention to reach their maximum at the insertion of the tracheal cannula. The increase observed in our study was similar to previously published data [15, 32] and is probably due to partial airway obstruction by the bronchoscope with concurrent hypoventilation [32, 33]. The marked drop in MV and compliance in the bronchoscopy group supports this theory. No similar hypercarbia was recorded in the VST group. Whereas PaCO_2_ values remained stable from the start of the intervention until tracheal cannulation, a further increase occurred until the placement of the tracheal cannula. We attribute this increase to the dilatation and occlusion of the trachea by the single-step dilator. However, hypercarbia in the VST group was significantly less pronounced than in the bronchoscopy group. Conversely, pH values decreased in both groups. Although remaining within normal limits in the VST group, the bronchoscopy group showed respiratory acidosis at the time of insertion of the tracheal cannula.

Although bronchoscopy is used worldwide in approximately 70% of all PDT procedures (and probably more often in Europe) [4], some authors advise against the routine use of bronchoscopy during PDT, owing to the risks of hypercarbia with consecutive respiratory acidosis, and endorse a risk-and-benefit assessment for each patient [6]. Data concerning hypercarbia during other tracheostomy techniques remain sparse, but better ventilation than with bronchoscopy-guided PDT has been described for surgical tracheostomy [32], whereas no data are available on the quality of ventilation during ultrasound-guided PDT or the landmark technique so far. Because the cross-sectional area of the endotracheal tube is not reduced in techniques without a bronchoscope, ventilation is believed to remain unchanged, except for the temporary occlusion due to the dilator prior to insertion of the tracheal cannula. Furthermore, a special double-lumen tube is available for guidance of PDT with ventilation via one lumen and bronchoscopy via the other [34]. The bronchoscopy lumen ends directly in front of the vocal cords, so that only the ventilation lumen with an inner diameter of 7.0 or 7.5 mm is passed through the vocal cords. This permits both visualization by bronchoscopy and sufficient ventilation. Data on this method remain sparse because it has so far been evaluated only in a ten-patient case-control study and should be further evaluated in the future [8]. Given the improved ventilation with the VST over bronchoscopy observed in our study, patients requiring stable ventilation (e.g., those with pulmonary hypertension [35] or decreased intracranial compliance after brain injury [36, 37]) may benefit from use of the VST.

Four patients in our study did not receive the assigned VST intervention, owing to technical problems. The reliability of the VivaSight^TM^ system consisting of camera, monitor, and the cable connection needs improvement. In one case, the camera could not be cleared of secretions through the rinsing channel after exchange of the endotracheal tube and therefore produced a blurred image that was insufficient to allow tracheostomy. In our experience, the camera needs to be cleared regularly after intubation with the VST, but so far no systematic data are available to indicate whether this is a common problem.

It should be noted that in experienced hands, use of bronchoscopy during PDT does not simply permit optical guidance by the visualization of the site of puncture and the opposite parts of the posterior tracheal wall. Unlike VST, bronchoscopy provides a high-resolution image and allows the bronchoscopist to inspect the more distal parts of the trachea. This may prevent minor tears in the mucosal lining of the posterior wall of the trachea following dilatation, which could progress to severe injury when inserting the tracheal cannula should it remain unnoticed. Furthermore, the use of bronchoscopy permits immediate intervention should complications, especially bleeding, arise; confirmation of the correct position of the tracheal cannula after its insertion; and clearance of blood and secretions from the bronchial tree.

Considering the costs of bronchoscopy vs. VST, the VST may offer cost savings compared with the visualization by bronchoscopy [12], but the costs of bronchoscopy depend on many factors (i.e., single-use vs. multiple-use bronchoscopes, frequency of uses per device, maintenance costs, and decontamination costs). Therefore, each institution should calculate the costs on the basis of their own data, including a surcharge for supplementary bronchoscopies in cases of insufficient visualization with the VST.

Our study has certain limitations. Our primary outcome measure was based on Likert scales. Although the ratings were based on objective parameters and obtained by an independent physician, the examiners’ expectations and opinions may have influenced the scoring, thus introducing a bias. Approximately one-third of the patients randomized to the VST group did not complete tracheostomy with the assigned intervention. This was not anticipated, and therefore the statistical power may have been reduced. However, we used ITT and PP analyses to reduce the risk of attrition bias and found similar results in both analyses.

In PDT with optical guidance using a VST, the endotracheal tube needs replacement before tracheostomy, which complicates the procedure and may pose a safety risk should the airway be lost. In our study, no desaturations or complications occurred related to the tube exchange; however, we excluded patients with a difficult airway. The manipulation of the VST to optimize the visualization of tracheal structures may increase the risk of tube displacement compared with bronchoscopy. We rated visualization and ventilation during PDT on 4-point Likert scales. Although this rating was based on objective parameters, we may have introduced bias when scoring the items.

## Conclusions

Visualization with the VST failed to show noninferiority to bronchoscopy; however, the site of cannulation could be identified with the VST and ventilation remained unchanged. Therefore, patients for whom normocapnia is essential (e.g., those with pulmonary hypertension or decreased intracranial compliance) may benefit from the use of the VST because this allows real-time visual guidance of tracheal cannulation without compromising the patient’s ventilation. Other patients presumably benefit from bronchoscopy because this technique allows for superior visualization. In light of the technical problems encountered in this study, we suggest that the VST device should be evaluated in a larger cohort of patients after improvement of its reliability.

## Additional files

Additional file 1: Table S1. Arterial blood gases and respiratory values (intention to treat analysis). (PDF 43 kb)

Additional file 2: Table S2. Analysis of score values. (PDF 36 kb)

Additional file 3: Figure S1. Rating of visualization and ventilation according to score (intention to treat analysis). (PDF 38 kb)

Additional file 4: Figure S2. Changes in paCO_2_ (intention to treat analysis). (PDF 40 kb)

Additional file 5: Figure S3. Changes in pH (intention to treat analysis). (PDF 40 kb)

## Abbreviations

ABG: Arterial blood gas
APACHE II: Acute Physiology and Chronic Health Evaluation II
C: Compliance
CONSORT: Consolidated Standards of Reporting Trials
etCO_2_: End-tidal carbon dioxide
FiO_2_: Fraction of inspired oxygen
Hb: Hemoglobin
ITT: Intention to treat
MAP: Mean arterial pressure
MV: Minute ventilation
n.s.: Not statistically significant
p.m.: Pars membranacea of the trachea
PaCO_2_: Partial pressure of arterial carbon dioxide
PaO_2_: Partial pressure of arterial oxygen
Paw: Airway pressure
PDT: Percutaneous dilatational tracheostomy
PEEP: Positive end-expiratory pressure
PP: Per protocol
RR: Respiratory rate
SaO_2_: Arterial oxygen saturation
SOFA: Sequential Organ Failure Assessment
SpO_2_: Oxygen saturation as measured by pulse oximetry
VST: VivaSight^TM^-SL endotracheal tube
V_T_: Tidal volume
