# Supplementary material for: Bronchoscopy versus an endotracheal tube mounted camera for the peri-interventional visualization of percutaneous dilatational tracheostomy - a prospective, randomized trial (VivaPDT)
Source: Crit Care. 2017 Dec 29;21:330. doi: 10.1186/s13054-017-1901-0 (PMC5747130; doi:10.1186/s13054-017-1901-0)
Supplement: Supplementary file 1 — Arterial blood gases and respiratory values (intention to treat analysis). (PDF 43 kb) [file 13054_2017_1901_MOESM1_ESM.pdf]

Additional File 1

Table S1: Arterial blood gases and respiratory values (intention to treat analysis)

	Timepoint 1		Timepoint 2			Timepoint 3		
	VivaSight™	Bronchoscopy	VivaSight™	Bronchoscopy	p	VivaSight™	Bronchoscopy	p
pH	7.47 [7.45;7.50]	7.44 [7.40;7.47]	7.44 [7.41;7.47]*	7.30 [7.27;7.34]*	< 0.001	7.37 [7.33;7.41]*#	7.26 [7.22;7.30]*#	< 0.001
paO_2 [kPa]*	11.1 [10.0;12.2]	11.0 [10.2;11.9]	53.8 [45.9;59.3]*	52.6 [44.7;60.5]*	ns	50.9 [42.1;58.3]*	51.3 [43.5;59.1]*	ns
$paCO_2$ [kPa]	5.0 [4.6;5.3]	5.3 [4.7;5.9]	5.4 [5.1;5.8]	7.6 [6.7;8.4]*	< 0.001	6.6 [5.9;7.0]*#	8.3 [7.2;9.5]*#	< 0.001
Lactat [mmol*L^{-1}]	0.9 [0.7;1.1]	1.0 [0.7;1.3]	0.8 [0.6;1.0]	0.9 [0.6;1.2]	ns	0.8 [0.6;1.0]	0.8 [0.6;1.1]	ns
Hb [g/dl]	8.8 [8.3;9.2]	9.0 [8.4;9.5]	8.6 [8.2;9.1]	8.9 [8.3;9.6]	ns	8.6 [8.2;9.0]	9.0 [8.3;9.6]	ns
S_aO_2 [%]	97 [96;98]	96 [96;97]	96 [88;105]	100 [100 –100]	ns	100 [99;101]	100 [100 –101]	ns
Paw [hPa]	21 [19;24]	21 [19;23]	23 [20;25]	23 [21;26]	ns	22 [21;25]	23 [21;25]	ns
PEEP [hPa]	7 [6;9]	7 [6;8]	7 [6;9]	7 [6;7]	ns	7 [6;9]	7 [6;7]	ns
$etCO_2$ [kPa]	4.9 [4.6;5.2]	4.6 [4.1;5.1]	4.7 [4.4;5.1]	4.5 [3.6;5.4]	ns	5.6 [5.0;6.2] #	6.4 [5.8;7.1]*#	0.031
V_t [ml]	436 [358;539]	446 [386;512]	476 [326;679]	250 [148;371]*	0.002	468 [382;598]	380 [257;465]	ns
MV [l*min^{-1}]	8.4 [7.3;9.5]	9.6 [8.0;11.3]	6.8 [5.4;8.3]	3.5 [2.5;4.8]*	0.001	9.2 [7.1;11.8]#	6.3 [4.8;7.8]*#	0.005
C [ml*hPa^{-1}]	53 [42;67]	57 [41;72]	32 [24;38]*	24 [7;34]*	ns	44 [29;60]	29 [22;38]*	0.05
RR [min^{-1}]	18 [16;20]	20 [17;23]	19 [17;20]	20 [18;22]	ns	19 [16;21]	21 [18;23]	ns
FiO_2	0.29 [0.25;0.31]	0.32 [0.25;0.39]	0.99 [0.97;1.01]*	0.99 [0.97;1.01]*	ns	0.99 [0.97;1.01]*	0.99 [0.97;1.01]*	ns
SpO_2 [%]	98 [97;99]	96 [95;98]	100 [100;100]*	100 [99;100]*	ns	100 [99;100]*	99 [99;100]*	ns
MAP [mmHg]	84 [76;92]	83 [76; 90]	92 [84;100]*	81 [74;88]	0.019	89 [82;96]	86 [81;93]	ns

Data are shown as the mean and 95% confidence intervals, timepoint 1: before start of intervention, timepoint 2: before tracheal cannulation, timepoint 3: after insertion of tracheal cannula, paO_2: arterial partial pressure of oxygen, $paCO_2$: arterial partial pressure of carbon dioxide, SaO_2: arterial saturation of oxygen, Paw: airway pressure, PEEP: positive end expiratory pressure, $etCO_2$: end tidal carbon dioxide tension, V_T: tidal volume, MV: minute ventilation, C: compliance, RR: respiratory rate, FiO_2: fraction of inspired oxygen, SpO_2: pulse oxymetric saturation of oxygen; statistical analysis was done with linear mixed models, p values in columns indicate difference between VivaSight and bronchoscopy groups at the respective timepoint; analysis of variables between timepoints: * $p < 0.05$ vs. timepoint 1, # $p < 0.05$ vs. timepoint 2, ns: not statistically significant.
