# Supplementary material for: Bronchoscopy versus an endotracheal tube mounted camera for the peri-interventional visualization of percutaneous dilatational tracheostomy - a prospective, randomized trial (VivaPDT)
Source: Crit Care. 2017 Dec 29;21:330. doi: 10.1186/s13054-017-1901-0 (PMC5747130; doi:10.1186/s13054-017-1901-0)
Supplement: Supplementary file 2 — Analysis of score values. (PDF 36 kb) [file 13054_2017_1901_MOESM2_ESM.pdf]

Additional File 2

Table S2: Analysis of score values

	ITT				PP			
score item	VST	bronchoscopy	mean difference	p	VST	bronchoscopy	mean difference	p
Visualisation	5.9 [4.7;7.1]	4.0[4.0;4.0]	1.9 [0.7;3.1]	0.003	5.4 [4.5;6.3]	4.0[4.0;4.0]	1.4 [0.5;2.3]	0.005
Ventilation	2.8 [2.3;3.3]	5.0 [4.4;5.7]	-2.3 [-3.0;-1.5]	<0.001	2.5 [2.1;2.9]	5.0 [4.4;5.7]	-2.5 [-3.2;-1.8]	<0.001
total	8.7 [7.6;9.8]	9.0 [8.4;9.7]	-0.3 [-1.6;0.9]	ns	7.9 [7.0;8.9]	9.0 [8.4;9.7]	-1.1 [-2.2;0]	ns
A	1.2 [1.0;1.5]	1.0 [1.0;1.0]	0.2 [-0.4;0.5]	ns	1.2 [0.9;1.5]	1.0 [1.0;1.0]	0.2 [-0.1;0.5]	ns
B	1.6 [1.3;.1.9]	1.0 [1.0;1.0]	0.6 [0.3;0.9]	<0.001	1.5 [1.3;1.8]	1.0 [1.0;1.0]	0.5 [0.2;0.8]	0.001
C	1.5 [1.1;2.0]	1.0 [1.0;1.0]	0.5 [0.1;1.0]	ns	1.2 [1.0;1.4]	1.0 [1.0;1.0]	0.2 [0;0.4]	ns
D	1.6 [1.2;2.0]	1.0 [1.0;1.0]	0.6 [0.2;1.0]	0.006	1.5 [1.2;1.8]	1.0 [1.0;1.0]	0.5 [0.2;0.8]	0.004
E1	1.3 [1.0;1.5]	2.4 [2.1;2.8]	-1.2 [-1.6;-0.7]	<0.001	1.2 [1.0;1.4]	2.4 [2.1;2.8]	-1.2 [-1.7;-0.8]	<0.001
E2	1.5 [1.2;1.8]	2.6 [2.3;3.0]	-1.1 [-1.5;-0.6]	<0.001	1.3 [1.1;1.6]	2.6 [2.3;3.0]	-1.3 [-1.7;-0.9]	<0.001

values are shown as mean and 95% confidence intervals in brackets; ITT: intention to treat analysis; PP: per protocol analysis; score items as given in table 1; analysis by Welch-test.
