# Supplementary material for: Bronchoscopy versus an endotracheal tube mounted camera for the peri-interventional visualization of percutaneous dilatational tracheostomy - a prospective, randomized trial (VivaPDT)
Source: Crit Care. 2017 Dec 29;21:330. doi: 10.1186/s13054-017-1901-0 (PMC5747130; doi:10.1186/s13054-017-1901-0)
Supplement: Supplementary file 3 — Rating of visualization and ventilation according to score (intention to treat analysis). (PDF 38 kb) [file 13054_2017_1901_MOESM3_ESM.pdf]

Additional File 3

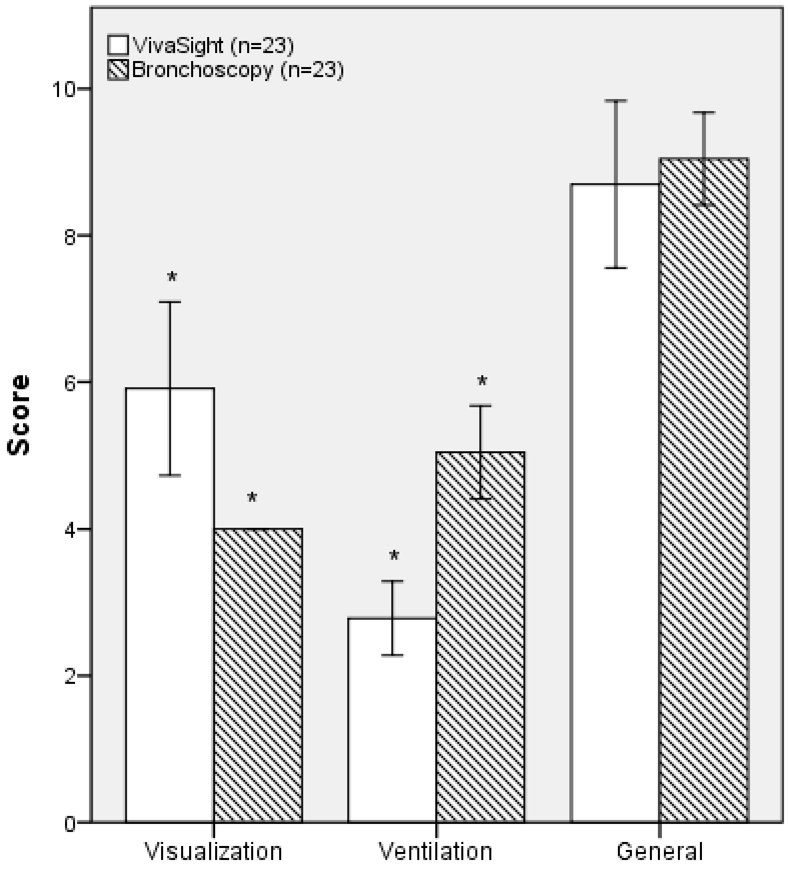

Figure S1: Rating of visualization and ventilation according to score (intention to treat analysis)

Lower scores indicate better performance. Error bars indicate 95% confidence intervals.
