# Supplementary material for: Bronchoscopy versus an endotracheal tube mounted camera for the peri-interventional visualization of percutaneous dilatational tracheostomy - a prospective, randomized trial (VivaPDT)
Source: Crit Care. 2017 Dec 29;21:330. doi: 10.1186/s13054-017-1901-0 (PMC5747130; doi:10.1186/s13054-017-1901-0)
Supplement: Supplementary file 4 — Changes in paCO2 (intention to treat analysis). (PDF 40 kb) [file 13054_2017_1901_MOESM4_ESM.pdf]

Additional File 4

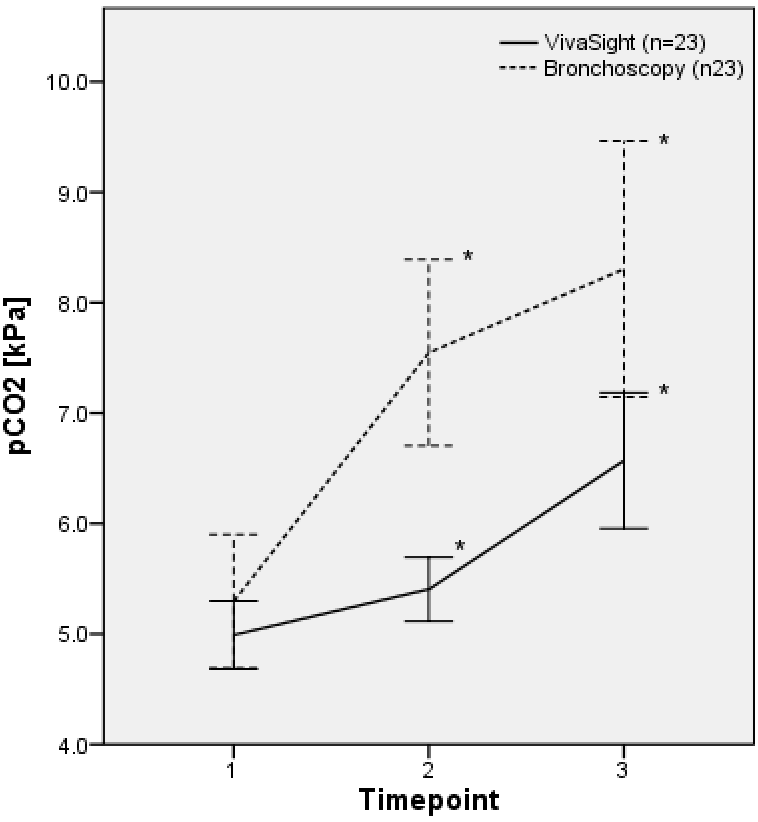

Figure S2: Changes in $paCO_2$ (intention to treat analysis)

Timepoint 1: before start of intervention, timepoint 2: before tracheal cannulation, timepoint 3: after insertion of tracheal cannula. Error bars indicate 95% confidence intervals. * $p<0.05$ for difference of groups at respective timepoint.
